# ZIPLA_S_: Zooplankton Index for Polish Lakes’ Assessment: a new method to assess the ecological status of stratified lakes

**DOI:** 10.1007/s10661-021-09390-7

**Published:** 2021-09-19

**Authors:** Agnieszka Ochocka

**Affiliations:** Department of Freshwater Protection, Institute of Environmental Protection-National Research Institute, Krucza 5/11D, 00-548 Warsaw, Poland

**Keywords:** Biological indicator, Crustacea, Ecological status assessment, Rotifera, Water Framework Directive

## Abstract

Zooplankton is widely recognized as a key component of pelagic ecosystems and forms the basis for major trophic webs. Although zooplankton has often been used as an indicator of trophic state, it has not been included as an obligatory element of the water assessment systems compliant with the Water Framework Directive. This article introduces the Zooplankton Index for Polish Lakes’ Assessment (ZIPLA_S_) as a new method to assess the ecological status of stratified lakes based on the zooplankton community. The ZIPLA_S_ evaluates three aspects of zooplankton communities, namely, taxonomic composition and abundance, diversity of the zooplankton community, and stressor-sensitive species, which are combined into a multimetric index. Following are the metrics used to compose multimetric ZIPLA_S_: percentage share of the Rotifer species indicative of high trophy in the indicative group’s number (IHTROT; %), ratio of Calanoida to Cyclopoida individual numbers (CA/CY), percentage of tecta form in the population of Keratella cochlearis (TECTA; %), Margalef’s index (d), and zooplankton abundance (NZOL; ind./L). ZIPLA_S_ responds clearly to eutrophication indicators—the strongest with Secchi disc visibility (Spearman’s rank correlation *R* = 0.86) and slightly weaker with the expressed by total phosphorus (*R* = -0.74), total nitrogen (R = 0.68) and the catchment pressure expressed by the nutrient loads generated by different types of land use (*R* = -0.58).

## Introduction

Zooplankton community is composed of small organisms (Crustacea and Rotifera) passively floating within the water column (or having only slight movement ability) inhabiting oceans, seas, and freshwaters, including lakes. Plankton animals play an important role in the functioning of aquatic ecosystems due to their position in the trophic chain. They are a valuable food source for planktivorous fish (top-down control) and feed on phytoplankton, controlling algae population (bottom-up control; Jeppesen et al., 2011).

Eutrophication is one of the major threats to European surface waters, including Poland. The initial stage of the eutrophication process stimulates the biological production and results in an increase in the number of fish. After exceeding a certain threshold of nutrient concentration, secondary effects of this process are observed. One of the most apparent effects of eutrophication is the massive development of planktic algae that creates mass blooms in the surface water layer, which reduces the water transparency (Lampert & Sommer, 2001) The limitation of light by phytoplankton causes the displacement of macrophytes and indirectly leads to the reconstruction of the composition of the accompanying fauna. In advanced eutrophication phase, oxygen depletion is usually observed in the bottom layer, which leads to the disappearance of fauna (e.g., sensitive relict species), including mass mortality of fish.

Eutrophication also affects the composition and abundance of zooplankton community. In the course of water nutrient enrichment, large Cladocerans are replaced by smaller ones (Jeppesen et al., 2000); this is the most evident effect of eutrophication. In pelagic zones of eutrophic lakes, small-bodied Cladoceran species such as *Bosmina* spp. and *Chydorus sphaericus* are generally more abundant than large-bodied species such as *Daphnia* spp. (DeMott & Kerfoot, 1982). Generally, low biomass of zooplankton is observed in oligotrophic lakes, which contain a great variety of species, while in lakes of advanced trophy, a large biomass with fewer species has been noted (Gannon & Stemberger, 1978). Under conditions of nutrient enrichment, the average size of zooplankton species often decreases, as smaller species with simpler life cycles and higher rates of reproduction become more abundant in the plankton community (Gliwicz, 1969). Zooplankton taxa have different preferences for trophic state (Berzins & Bertilsson, 1989; Berzins & Pejler, 1989; Lougheed & Chow-Fraser, 2002) and water clarity. Obviously, many species occur in lakes with various trophies, but within Crustacean and Rotifer communities, some species prefer high or low trophic waters.

Deterioration of the conditions in a lake adversely impacts features of zooplankton community; for example, it leads to increased biomass and abundance (Hanson & Peters, 1984), decrease in body size (Karpowicz et al., 2020; Pace, 1986), and reduction in species diversity (Andronikova, 1996; Haberman & Haldna, 2014). Therefore, zooplankton could be a potentially effective indicator to assess small changes in water quality, especially those caused by eutrophication. Comprehensive studies demonstrate the use of zooplankton as an effective eutrophication indicator (Andronikova, 1996; Carpenter et al., 2006; Čeirāns, 2007; De-Carli et al., 2019; Dembowska et al., 2015; Ejsmont-Karabin, 2012; Ejsmont-Karabin & Karabin, 2013; Haberman & Haldna, 2014; Karabin, 1985; Karpowicz et al., 2020; Ochocka & Pasztaleniec, 2016). Nevertheless, these research studies address trophic categories provided by the Organisation for Economic Cooperation and Development (OECD, 1982), not ecological status classes sensu the Water Framework Directive (WFD, European Commission (EC), 2000).

The WFD introduced a new approach to water quality assessment, which does not refer directly to traditional trophic categories. It refers to the concept of ecological status, understood as ecosystem health, and departs from the traditional approach that employs static water trophic categories. In this concept, naturally eutrophic conditions (without the influence of anthropogenic pressure) are considered as an acceptable state, while eutrophication refers to undesirable effects of nutrient load, resulting from anthropogenic pressure (Soszka, 2009). Consequently, mesotrophic lake, when anthropogenically impacted and altered in relation to natural status, may represent deteriorated ecological status (worse than good), whereas eutrophic lake slightly deviated from natural conditions may represent good ecological status. Thus, while assessing ecological status, it is pivotal to establish type-specific reference conditions, which constitute a benchmark for evaluation of the deviation of the current state from the state expected under undisturbed conditions.

The WFD operates with five classes of ecological status, assessed by using the following biotic elements: phytoplankton, macrophytes and phytobenthos, invertebrates, and fish, and supporting physicochemical and hydromorphological elements. These elements help assess primarily the impact of eutrophication on aquatic ecosystems, and, to a lesser extent the hydromorphological pressure (Poikane et al., 2020). During the last two decades, in all the EU countries, new type-specific WFD-compliant biological methods of ecological status assessment have been elaborated on. In Poland, lake assessment methods based on phytoplankton (Hutorowicz & Pasztaleniec, 2014), macrophytes (Ciecierska & Kolada, 2014), phytobentos (Zgrundo et al., 2020), macroinvertebrates (Bielczyńska et al., 2020), and ichthyofauna (Adamczyk & Prus, 2020) have been implemented under the purview of state monitoring program. However, zooplankton has not been included as one of the obligatory biological quality elements recommended in the WFD. The only mention of this biological element appears in the WFD Monitoring Guidelines elaborated by Working Group within the Common Implementation Strategy (CIS, 2003) supporting WFD implementation, where its analysis has been limited to the role of a “supporting/interpretative parameter” in the assessment of lakes based on fish assemblages.

The reason for this omission remains unclear (Caroni & Irvine, 2010), particularly as zooplankton, has been traditionally involved in the ecological research of lakes in many places, in Russia (Andronikova, 1996), North America (Kane et al., 2009), and in Europe, e.g., Poland (Karabin, 1985; Radwan & Popiołek, 1989), Czechoslovakia (Sládeček, 1983), Sweden (Pejler, 1983), Finland (Hakkari, 1972), the Netherlands (Gulati, 1983), Denmark (Jeppesen et al., 2000), Estonia (Haberman & Haldna, 2014), and Greece (Stamou et al., 2019). Moreover, zooplankton has been used for decades as a bioindicator for routine lake monitoring in Austria, Denmark, Finland, the Netherlands, and Norway (European Environment Agency (EEA), 1996). Zooplankton seems to be a promising indicator for the assessment of the ecological status of lake ecosystems due to its vulnerability to the effects of anthropogenically induced eutrophication and the relatively easy determination of species in contrast to phytoplankton (Ejsmont-Karabin, 2012; Ochocka & Pasztaleniec, 2016).

The failure to include zooplankton as part of the assessment of the ecological status of lakes has been emphasized by Moss (2007). In the scientific literature, the need for the inclusion of zooplankton in the assessment of lake water quality is gaining increased attention (Caroni & Irvine, 2010; Ejsmont-Karabin, 2012; Jeppesen et al., 2011; Karpowicz et al., 2020; Ochocka & Pasztaleniec, 2016).

This study aimed to develop a zooplankton-based index and assessment system to evaluate the ecological status of deep, stratified lakes, in the context of the WFD approach. The present work comprises (1) the selection of candidate zooplankton metrics and their testing in a pressure gradient; (2) the establishment of reference conditions for temperate lowland, stratified lakes; (3) the development of the multimetric index ZIPLA_S_; (4) testing of the multimetric’s response along the pressure gradient (eutrophication indicators); and (5) setting of the class boundary values for ZIPLA_S_. Since no specific guidelines for elaborating zooplankton multimetric exists, general guidelines for defining biological WFD-compliant metrics and the criteria for the selection of multimetric components for aquatic invertebrates were adopted (Hering et al., 2006). The ZIPLAs multimetric consists of five metrics that take into account the composition and abundance and the diversity and occurrence of sensitive taxa. Compared to single-metrics indices, multimetric indices act as a complex tool for assessing water ecosystems, since they integrate different stressors and components of the community (Hering et al., 2006). To meet the criteria for a reliable assessment metric, an index should respond significantly and directionally to pressure.

## Material and methods

### Study area

**Fig. 1:**
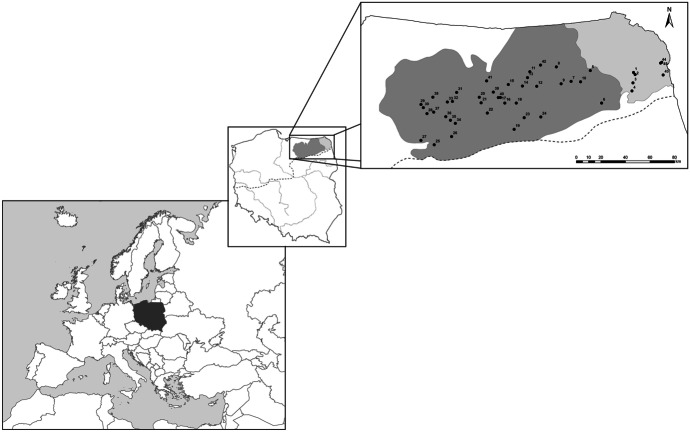
Localization of studied lakes in Masurian (dark gray color) and Lithuanian (light gray color) Lakelands. The black, dotted line shows the area of the last Baltic glaciation; the gray line shows the largest rivers in Poland. The numbers refer to the lake names: 1—Blizno; 2—Busznica, 3—Kalejty, 4—Sajno; 5—Olecko Małe; 6—Rajgrodzkie; 7—Łaśmiady; 8—Gawlik; 9—Garbaś Mały; 10—Zdrężno; 11—Niegocin; 12—Buwełno; 13—Boczne; 14—Jagodne; 15—Ryńskie; 16—Majcz Wielki; 17—Kuc; 18—Mikołajskie; 19—Nidzkie; 20—Lampackie; 21—Piłakno; 22—Gant; 23—Jegocin; 24—Roś; 25—Omulew; 26—Świętajno; 27—Maróz; 28—Bartąg; 29—Ukiel; 30—Kortowskie; 31—Dadaj; 32—Tumiańskie; 33—Kierźlińskie; 34—Leleskie; 35—Kalwa; 36—Purda; 37—Linowskie; 38—Wadąg; 39—Czos; 40—Probarskie; 41—Kiersztanowskie; 42—Kruklin; 43—Brożane; 44—Wiłkokuk; 45—Zelwa

Zooplankton samples were collected from 45 lakes located in north-eastern Poland (see Fig. 1) during the summer period (July–August), of the years 2012–2015 while conducting the dedicated research projects.

Seven lakes were investigated three times, 27 lakes were inestigated two times, and 11 lakes were investigated once during this period, resulting in 86 lake-years including repetitions. In the temperate zone, the summer stagnation is a stable period when changes in the abiotic and biotic environmental conditions are less. During this period, zooplankton communities are most diverse and attain the highest abundance level (Karabin, 1985). All of the analyzed lakes are lowland (< 200 m a.s.l.), with a surface area ranging from 0.391 to 26 km^2^ and with alkaline water (> 1.0 meq/L). They are deep, stratified water bodies with a mean depth ranging from 4 to 13 m and a maximum depth ranging from 12 to 57 m (see Table SI 1).

### Data collection

The sampling points were located close to the deepest part of each lake. The samples for chemical and zooplankton analyses were taken using a 2.6-L Limnos sampler at intervals of 1-m depth from the surface to the bottom of the epilimnion layer. Water was filtered using a plankton net with a 30-µm mesh size and preserved with Lugol’s solution and 4% formalin. Secchi disc visibility (SD) was measured and field measurements of water temperature, pH, conductivity, and oxygen concentration were carried out using a YSI 6600 V2 multiparametric probe (Ohio, USA). The chemical analyses of total phosphorus (TP) and total nitrogen (TN) concentration were performed in a laboratory, using standard methods (Hermanowicz et al., 1999). The measurement of chlorophyll *a* concentration was performed by a spectrophotometric method (Nusch, 1980).

Cladocerans and Rotifers were identified to species level. Copepods were divided into nauplii and copepodites, which were identified to order level, while adult copepods were identified to species level. The Crustacean zooplankton biomass was estimated based on the relationship between the body length and body weight for each species, as proposed by Balushkina and Vinberg (1979). The standard wet weight of Rotifers was determined from the individual body weights, as suggested by Ejsmont-Karabin (1998). The species *Asplanchna priodonta* and *Leptodora kindtii* were excluded from the analysis because of their large size, which was many times greater than that of both Rotifer and Crustacean species.

### Elaboration of the new zooplankton method

The essential step in elaborating the WFD-compliant ecological status assessment system is to establish reference conditions. Reference lakes were defined based on spatially approach (“the best of existing”), where data from undisturbed or minimally disturbed lakes with only slight human disturbances are analyzed. This approach is among the ones recommended by the WFD and has been used in other studies of such kind (e.g., Birk et al., 2012; Lyche-Solheim, 2005; Soszka et al., 2008).

To assign a lake as a reference, the following criteria were applied:

-no point sources of pollution in the total catchment.

-natural land use in the catchment (> 80% area of forests or wetlands, lack of villages in direct contact with the shoreline, no urban areas)

-lack or no intensive recreational use.

-high/good water quality according to existing data.

The data on water quality indicators, which were used to select the reference lakes, came from the Polish State Environmental Monitoring (SEM) program; these data were obtained in the years 2009–2012.

The impact of catchment use on the quality of lake waters was analyzed based on the CORINE Land Cover 2018 (CLC18; Büttner & Kosztra, 2017). The area (km^2^) occupied by various forms of land use was calculated for each lake. Further, theoretical loads of nitrogen and phosphorus generated by individual forms of land use were calculated using unit values of surface runoff for the individual land use category in the total catchment (Arciszewski et al., 2010). The values of unit loads are presented in Table 1.

**Table 1:** The values of unit loads of nutrients depend on type of land use (after Arciszewski et al., 2010)

Type of land use	Unit loads (kg/ha/year)	
	*N*	*P*
Forests	1.5	0.1
Agricultural areas and discontinuous urban fabric	9.0	0.3
Pastures	3.0	0.2
Wetlands	1.5	0.1
Land principally occupied by agriculture with significant areas of natural vegetation	3.0	0.2
Continuous urban fabric	6.0	0.9

To quantify the pressure caused by different forms of land use in the catchment area, for each lake, the cumulative nutrient load index (PCA_TOT_) was calculated, based on the principal component analysis performed in the MVSP software (Kovach, 2007). The PCA_TOT_ index used the values of correlation coefficients of the first component axis (PC1) from theoretical TP and TN loads, calculated per unit of water volume (P/V, N/V; Kutyła, 2020). Ultimately, TP, TN, and SD as well as the PCA_TOT_ were adopted as parameters of pressure proxies. Based on extensive literature reviews (Andronikova, 1996; Ejsmont-Karabin, 2012; Ejsmont-Karabin & Karabin, 2013; Karabin, 1985; Karpowicz et al., 2020; Margalef, 1958; Shannon & Weaver, 1963), a list of 31 candidate zooplankton indices was selected, which can be sub-divided into three groups based on following characteristics: (1) the composition and abundance of fauna, (2) the diversity of the zooplankton community, and (3) occurrence of sensitive taxa (see Table 2). These indices were tested against proxies of eutrophication parameters (TP, TN, SD) for their response to eutrophication pressure, and best responding metrics within each group were selected to compose the multimetric index (see Table SI 2).

**Table 2:** Overview of zooplankton indices tested to develop ZIPLA_S_ multimetric

Index type	Acronym	Description	Unit	References	Crustacea/Rotifera
Composition/abundance index	NCRU	Numbers of Crustacea [ind./L]	ind./L	Karabin (1985), Ejsmont-Karabin and Karabin (2013)	Crustacea
	BCL	Biomass of Cladocera	mg w. wt./L		Crustacea
	BCY	Biomass of Cyclopoida	mg w. wt./L	Karabin (1985), Ejsmont-Karabin and Karabin (2013)	Crustacea
	BCA	Biomass of Calanoida	mg w. wt./L		Crustacea
	BCRU	Biomass of Crustacea	mg w. wt./L		Crustacea
	CB	Percentage of cyclopoid biomass in total biomass of Crustacea	%	Karabin (1985), Ejsmont-Karabin and Karabin (2013)	Crustacea
	CY/CL	Ratio of Cyclopoida biomass to the biomass of Cladocera	mg w. wt./L		Crustacea
	CL/CY	Ratio of Cladocera biomass to the biomass of Cyclopoida	mg w. wt./L		Crustacea
	CA/CY	Ratio of Calanoida to Cyclopoida individual numbers	ind./L		Crustacea
	CY/CA	Ratio of Cyclopoida to Calanoida individual numbers	ind./L	Karabin (1985), Ejsmont-Karabin and Karabin (2013)	Crustacea
	B/NCRU	Ratio of biomass to numbers	mg w. wt./L ind./L	Karabin (1985), Ejsmont-Karabin and Karabin (2013)	Crustacea
	ND/NCRU	Ratio of *Daphnia* to Crustacea numbers	ind./L		Crustacea
	CL/Cop	Ratio of Cladocera to Copepoda (Cyclopoida + Calanoida) numbers	ind./L	Andronikova (1996)	Crustacea
	NROT	Rotifera numbers	ind./L	Ejsmont-Karabin (2012)	Rotifera
	BROT	Biomass of Rotifera	mg w. wt./L	Ejsmont-Karabin (2012)	Rotifera
	B/NROT	Ratio of biomass to numbers	mg w. wt./L ind./L	Ejsmont-Karabin (2012)	Rotifera
	BMA	Macrozooplankton biomass	mg w. wt./L		Crustacea/Rotifera
	BME	Mesozooplankton biomass	mg w. wt./L		Crustacea/Rotifera
	BMI	Microzooplankton biomass	mg w. wt./L		Crustacea/Rotifera
	NCRU/NROT	Ratio of Crustacea to Rotifera numbers	ind./L		Crustacea/Rotifera
	BCRU/BROT	Ratio of Crustacea to Rotifera biomass	mg w. wt./L	Andronikova (1996)	Crustacea/Rotifera
	NZOL	Zooplankton abundance	ind./L		Crustacea/Rotifera
	Nsp	Species number	ind./L		Crustacea/Rotifera
	BZOL	Zooplankton biomass	mg w. wt./L		Crustacea/Rotifera
Sensitivity index	IHTCRU	Percentage of species indicative of high trophy in the indicative group's numbers	%	Karabin (1985), Ejsmont-Karabin and Karabin (2013)	Crustacea
	TECTA	Percentage of form tecta in the population of Keratella cochlearis	%	Ejsmont-Karabin (2012)	Rotifera
	IHTROT	Percentage of species indicative of high trophy in the indicative group's number	%	Ejsmont-Karabin (2012)	Rotifera
Functional index	Dc_bl	*D. cucullata* body length	µm	Karpowicz et al. (2020)	Crustacea
	BAC	Percentage of bacteriovores in total rotifer numbers	%	Ejsmont-Karabin (2012)	Rotifera
Diversity index	d	Diversity index		Margalef (1958)	Crustacea/Rotifera
	H'	Diversity index		Shannon and Weaver (1963)	Crustacea/Rotifera

Both the pressure parameters and the tested zooplankton indices did not show a normal distribution; therefore, for all the analyses, the non-parametric statistical tests were used. To select indicators that are sensitive to the intensity of eutrophication, the relationship between the values of the indicators and the parameters of water quality was investigated using Spearman’s rank correlation coefficient method. The indicators that responded well to pressure were those that first showed a statistically significant correlation with the concentration of TP, which is commonly considered as the basic indicator of eutrophication (Lyche-Solheim et al., 2013) and with which the parameters of the zooplankton community are often correlated (Jeppesen et al., 2011; Sondergaard et al., 2005). The threshold value of Spearman’s correlation coefficient (to consider a metric as the one responding well to pressure) was set as *R* > 0.55. Among the 31 tested indices, those that most strongly correlated with pressure (*R* > 0.55; see Table SI 2) and represented all metric groups were considered as components of the ZIPLA_S_ multimetric index. The Water Framework Directive introduced the concept of a “metric,” so in this work, indices selected to create the ZIPLAs multimetric were referred as „metrics”. The multicollinearity among the selected metrics was assessed by examining tolerance and variance inflation factor (VIF). The values of zooplankton metrics (which have different units) selected for use in the ZIPLA_S_ were normalized to ecological quality ratios (EQRs), ranging from 0 (the worst status) to 1 ( the best status), using the following equation (Hering et al., 2006):

For indices decreasing with increasing pressure:

$$\mathrm{EQR}=(\mathrm I\mathrm n\mathrm d\mathrm{ex}\_\mathrm{re}\mathrm s\mathrm u\mathrm l\mathrm t-\mathrm L\mathrm o\mathrm w\mathrm{er}\_\mathrm{An}\mathrm c\mathrm h\mathrm o\mathrm r)/(\mathrm{Upper}\;\_\mathrm{An}\mathrm c\mathrm h\mathrm o\mathrm r-\mathrm L\mathrm o\mathrm w\mathrm{er}\_\mathrm{An}\mathrm c\mathrm h\mathrm o\mathrm r)$$

For indices increasing with increasing pressure:

$$\mathrm{EQR}=1-(\mathrm{Index}\_\mathrm{result}-\mathrm{Lower}\_\mathrm{Anchor})/(\mathrm{Upper}\;\_\mathrm{Anchor}-\mathrm{Lower}\_\mathrm{Anchor})$$

Values > 1 were set to 1, while values < 0 were set to 0.

To analyze the response of the selected indices to eutrophication expressed by TP concentration, scatter plots based on lowess smoothed models were used.

The ZIPLA_S_ multimetric is the arithmetic mean of the values of its compositional metrics.

The boundary values for five ecological status classes, i.e., high (H), good (G), moderate (M), poor (P), and bad (B), were determined based on the distribution of ZIPLA_S_ values in the studied lakes. The high/good class boundary (H/G) was set as the 25th percentile of ZIPLA_S_ values for reference lakes, as recommended by Hering et al. (2006). The other boundaries were established using the subsequent percentages of the H/G limit value of ZIPLA_S_: 75% for G/M, 50% for M/P, and 25% for the P/B boundary. The performance of the ZIPLA_S_ along nutrient gradients was tested using Spearman’s rank correlation test. To show the statistical differentiation of ZIPLA_S_ among ecological classes, the non-parametric Mann–Whitney *U* test was conducted. All statistical analyses were carried out using STATISTICA 12.0 PL software (StatSoft Inc., 2014).

## Results

### Environmental characteristics

The parameters of water quality in analyzed lakes indicated trophic conditions, ranging from mesotrophy to hypertrophy (see Table SI 1). In 11 of the 45 lakes, the summer TP values exceeded 60 ug/L, indicating high fertility and hypertrophic conditions, which was reflected in the low values of SD, ranging from 0.8 to 1.8 m. In seven of the least fertile lakes, the TP concentration was less than 20 ug/l, and the chlorophyll content ranged from 1.9 to 10.8 ug/L. These lakes had the highest SD, ranging from 3 to 7.2 m.

### Catchment impact

The total catchment area of the examined lakes ranged from 3.6 to 30,303.0 km^2^. In the case of 12 lakes, the total catchment area was forested by more than 50%. For seven of these lakes, this share exceeded 80% of the catchment area. The analyzed pool of lakes included 12 lakes located in agricultural catchments, where arable land occupied more than half of the total catchment area. The use of the catchment area of other lakes was diversified without a clear dominance of one of the analyzed categories of land use. The values of theoretical phosphorus loads generated in the catchments ranged from 63.0 to 51,487.0 kgP/year and nitrogen loads from 1550.0 to 1,226,983.0 kgN/year. The total catchment area and its type of use influenced the size of nutrient loads entering the lakes.

The phosphorus load potentially generated in the total catchment per unit of water volume ranged from 0.003 to 1.090 gP/m^3^, and the nitrogen load ranged from 0.07 to 26.70 gN/m^3^. The values of the PCA_TOT_ index ranged from -0.33 (low pressure) to 2.60 (high pressure).

### Reference conditions

Six of 45 analyzed lakes were indicated as references based on the pressure criteria. Maximum depth of lakes that were designated as reference ranged from 12 to 48 m, while mean depth ranged from 3.8 to 9 m. Natural land use in the total catchments area ranged from 91 to 100%. Considering the trophic parameters, for the majority of lakes, the concentration of TP rarely exceeded 30 ug/l. Lakes Zelwa and Wiłkokuk investigated in 2013 were exceptions, where higher values have been reported: 46 ug/l and 54 ug/l, respectively. TN concentrations ranged from 0.15 to 0.94 mg/l. Transparency expressed by SD ranged from 2 to 7 m (see Table SI 1). In all reference lakes, 65 species of zooplankton were identified, among which, 28 belonged to Crustacea and 36 to Rotifera. In the Crustacean community, Cladocera was the dominant group. The most frequent species among Crustaceans were *Diaphanosoma brachyurum* (18%), *Daphnia cucullata* (13%; which is an indicator species of low-trophic lakes), *Eubosmina crassicornis* (11%), and *Eudiaptomus graciloides* (11%). Following species are typical for low-trophic polish lakes—*Daphnia cristata*, *Daphnia galeata*, *Daphnia hyalina*, *Eubosmina coregoni*, *Bythothrepes longimanus,* and *Heterocope appendiculata*—were found in waters of these lakes (Ejsmont-Karabin & Karabin, 2013). In the Rotifer community, *Keratella cochlearis* occurred most frequently (40%), which is a species commonly found in all types of water. Low-trophic species had a large share in the abundance of Rotifer community: *Polyarthra major* (11%), *Conochilus unicornis* (5%), *Gastropus stylifer* (5%), and *Ascomorpha ecaudis* (3%).

### Development of a multimetric index

Among all of the tested indices, five among the ones that were most strongly correlated with TP, TN, SD, and PCA_TOT_ were selected (see Table 3).

**Table 3:** Spearman’s rank correlation coefficients between proxies of eutrophication (TP—total phosphorus, TN—total nitrogen, SD—Secchi disc visibility) as well as index of anthropogenic pressure (PCA_TOT_—the cumulative nutrient load factor) and selected metrics

Index type	Acronym	Correlations with trophy parameters							
		TP		TN		SD		PCA_TOT_	
		*r*	*p*	*r*	*p*	*r*	*p*	*r*	*p*
Composition/abundance index	CA/CY	-0.63	< 0.001	-0.51	< 0.001	0.77	< 0.001	-0.392	< 0.001
	NZOL	0.56	< 0.001	0.61	< 0.001	-0.75	< 0.001	0.564	< 0.001
Sensitivity index	TECTA	0.61	< 0.001	0.73	< 0.001	-0.85	< 0.001	0.644	< 0.001
	IHTROT	0.67	< 0.001	0.70	< 0.001	-0.75	< 0.001	0.555	< 0.001
Diversity index	d	-0.61	< 0.001	-0.49	< 0.001	0.66	< 0.001	-0.373	< 0.001

These metrics have different ranges of values and different directions of action; therefore, before they were combined in the multimetric index, their values were normalized according to the formulas given in Eqs. 1–5:

1 $$\mathrm{EQR CA}/\mathrm{CY}=\frac{[\mathrm{CA}/\mathrm{CY}-0.0035]}{\mathrm{1,4340}}$$

2 $$\mathrm{EQR NZOL}=1-\frac{\left[\mathrm{NZOL}-194.7000\right]}{2287.2500}$$

3 $$\mathrm{EQR TECTA}=1-\frac{[\mathrm{TECTA}-0.0000]}{78.6680}$$

4 $$\mathrm{EQR IHTROT}=1-\frac{[\mathrm{IHTROT}-\mathrm{0,0000}]}{100.0000}$$

5 $$\mathrm{EQR d}=\frac{[\mathrm{d}-2.5510]}{3.4520}$$

**Fig. 2:**
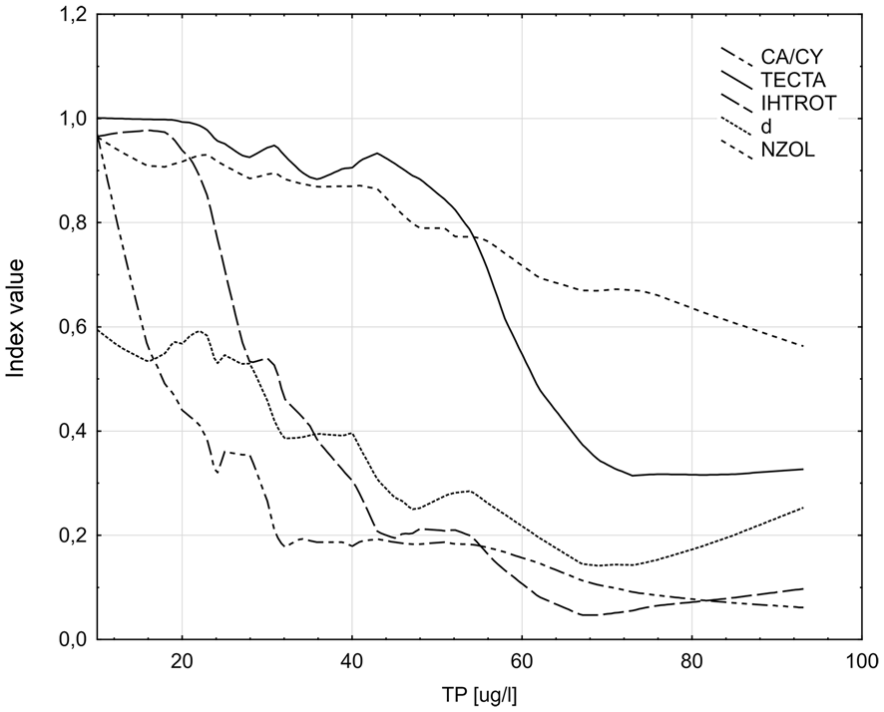
Relationship between normalized zooplankton indices selected to develop ZIPLAs multimetric and total phosphorus concentrations, lines represent the lowess smoothed models

The response of normalized values of these indices (ranging from 0 to 1) to TP concentration between 10 and 100 µg/L varied considerably (see Fig. 2).

The lowess smoothed model regression lines showed nonlinearity for five metrics in the analyzed spectrum of TP; only in the case of NZOL, the relationship approximated the linear model. Below the concentration of 10 µgP/L most indices were close to 1.0, indicating reference conditions, and only Margalef’s index reached a value of 0.6. In the TP range from 10 to 30–45 µg/L, the values of CA/CY and IHTROT decreased rapidly from 1.0 to 0.2, whereas in higher TP concentrations, no response was observed. The values of NZOL gradually decreased from 1.0 to 0.6 with increasing TP concentration, throughout the trophic gradient. The values of Margalef’s index systematically decreased (within the range from 0.6 to 0.2) in the TP ranging from 10 to 70 µg/L. Beyond TP value of 70 µg/L, the curves of most indices (except for NZOL) flattened out but at different levels, in the range from 0.3 to below 0.1. TECTA values slightly changed at low TP concentrations (below 45 µg/L), whereas at more than 45 µg/L, the values decreased rapidly (from about 0.9 to 0.3) until the threshold value of 70 µg/L was reached.

The normalized values of selected metrics were combined into ZIPLA_S_ multimetric index (arithmetic average; Eq. 6):

6 $${\mathrm{ZIPLA}}_{\mathrm{s}}=\frac{\mathrm{CA}/\mathrm{CY}+\mathrm{NZOL}+\mathrm{TECTA}+\mathrm{IHTROT}+d}{5}$$

**Fig. 3:**
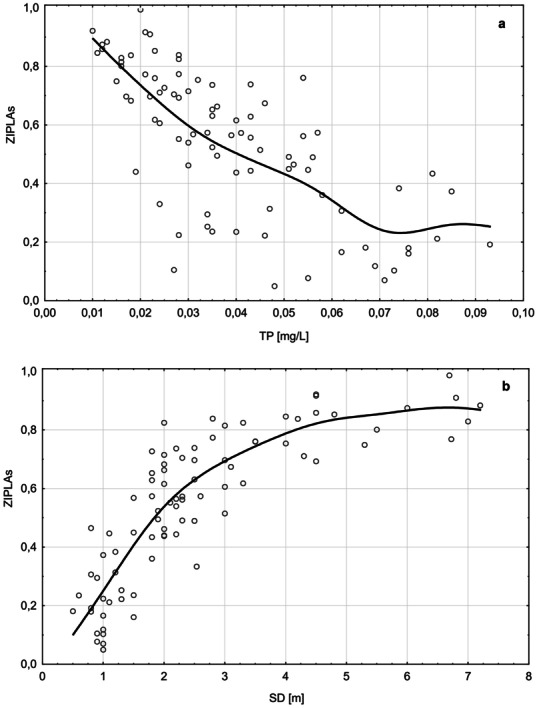
Relationships between ZIPLA_S_ and TP (**a**) and SD (**b**) in 45 lakes surveyed in the years 2012–2015. The *lines* represent the distance weight least squares smoothing fitted model

The ZIPLA_S_ showed strong correlations with all pressure parameters—tested, the strongest and positive correlations were observed with SD (*R* = 0.86; *p* < 0.0001), while slightly weaker and negative correlations with TP, TN, and PCA_TOT_ (*R* = -0.74, *R* = -0.68 and *R* = -0.58; *p* < 0.0001, respectively). The relationship between the ZIPLA_S_ multimetric and selected proxies of eutrophication (TP and SD) are shown in Fig. 3.

### Boundary setting of ecological status classes.

The distribution of ZIPLA_S_ values in investigated lakes was analyzed to determine boundary values for the ecological status classes. The H/G class boundary was thus set at 0.755. In the other classes, boundaries were set by dividing the range of ZIPLA_S_ values between the H/G boundary into four (see Table 4).

**Table 4:** Boundary values of ZIPLA_S_ for ecological status classes

Ecological status	Range of ZIPLA_S_ values
High	≥ 0.755
Good	0.566–0.754
Moderate	0.377–0.565
Poor	0.189–0.376
Bad	≤ 0.189

Based on the developed boundaries of ZIPLA_S_ classes, 20 lake-years were assessed as high, 25 as good, 18 as moderate, 13 as poor, and 10 as bad.

**Fig. 4:**
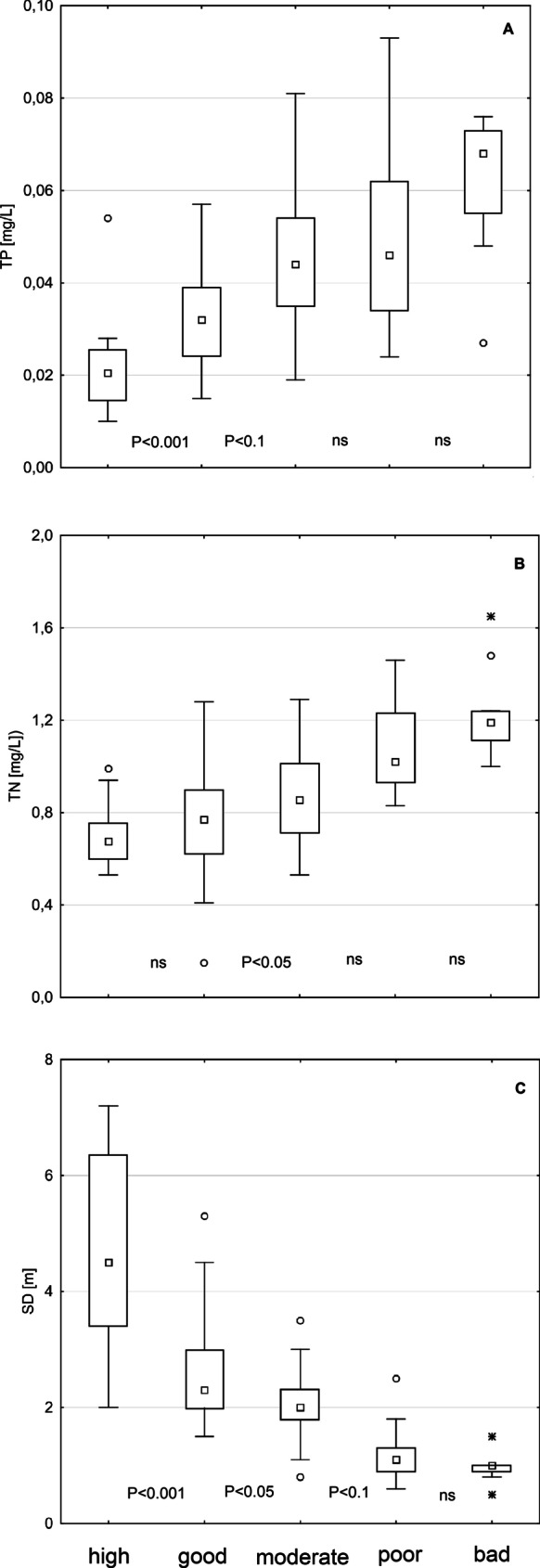
Distribution of TP (**A**), TN (**B**), and SD (m) (**C**) in lakes classified to one of the five classes of ecological status according to the ZIPLA index. High (*N* = 20), Good (*N* = 25), Moderate (*N* = 18), Poor (*N* = 13), Bad (*N* = 10); Boxplots: 25–75th percentiles with median, whiskers: range, circles: outliers, stars: extreme values. The level of confidence in comparison of distribution of nutrients between subsequent classes obtained in Mann–Whitney *U* test

The distribution of ZIPLA_S_ values across ecological status classes differed significantly (see Fig. 4).

For all water quality parameters, ZIPLA_S_ differentiated between good and moderate classes, whereas in the best classes (high and good), differentiation was found for TP and SD. In the worst status, a clear overlap between poor and bad classes for all eutrophication indicators (TP, TN, SD) was noticed.

## Discussion

Based on the physicochemical analysis, investigated lakes represented a varied spectrum of trophic conditions that are typical for Polish lakes (Siuda et al., 2013; Zdanowski, 1983). According to the WFD, the methods of assessing the ecological status should evaluate not only the quality of the water, but also the degree of deviation from conditions not disturbed by human activity. Among the analyzed lakes, six met the criteria of the reference lakes. This is crucial in understanding the role of zooplankton in assessing the ecological status of lakes, as so far, zooplankton has been used frequently in assessing trophic conditions and the difference between trophic and ecological status, which still remains unclear. Previous studies show that Rotifer indices are the best for the assessment of trophic status (Ejsmont-Karabin, 2012; Ferdous & Muktadir, 2009; Karabin, 1985). Both Crustacean and Rotifer indices were tested, since the literature also shows that Calanoida, which prefer low trophic conditions, may be good indicators of even a slight deterioration of the water quality in low-trophic lakes (Gannon & Stemberger, 1978).

For the development of the ZIPLA_S_ multimetric index, metrics that correlated most strongly with all pressure indicators and reflected various aspects of the zooplankton community were selected. In total, five component metrics were selected as base for ZIPLA_S_ multimetric development:

The percentage share of the Rotifer species indicative of high trophy in the indicative group’s number (IHTROT; %) showed the strongest correlation with the proxies of eutrophication. Indicator Rotifer species, typical for high-trophic lakes in Poland, are listed by Ganonn and Stemberger (1978) as indicator species of high-trophic lakes in North America. However, some morphological differences among species may exist even on the same continent. Therefore, using this index, region-specific list of indicator species for low and high trophy should be developed for different countries. Based on the research by Ejsmont-Karabin (2012) and Karabin (1985), the following species have been assigned as indicators of high trophy: *Keratella cochlearis* f. *tecta*, *Keratella quadrata*, *Pompholyx sulcata*, *Filinia longiseta*, *Anuraeopsis fissa*, *Trichocerca pusilla*, *Brachionus angularis*, and *Brachionus diversicornis*. Moreover, following were the indicators of low trophy: *Ascomorpha ovalis*, *Conochilus hippocrepis*, *Ascomorpha ecaudis*, *Gastropus stylifer*, and *Polyarthra major*.

The ratio of Calanoida to Cyclopoida individual numbers (CA/CY) was the only Crustacean index and the second strongest correlating index with TP. The value of this index decreases with increasing eutrophication. Based on the obtained results, it was found that the abundance of Calanoida decreases with an increase of trophy, while the abundance of Cyclopoida increases. This confirms the results of the research by Gannon and Stemberger, (1978), which showed that Calanoida prefers oligotrophic waters, where they are much more abundant, compared to waters of high trophy. These authors, based on the research of the American Great Lakes, concluded that *Limnocalanus macrurus* and *Senecella calanoides* belonging to the order Calanoida are effective indicators of low trophic waters, as they prefer cool, well-oxygenated waters.

Percentage of *tecta* form in the population of *Keratella cochlearis* (TECTA; %): Hillbricht-Ilkowska (1972) and Peljer (1962) show that in eutrophic lakes, *Keratella cochlearis* reaches a smaller body size and the length of their posterior spine is less than those inhabiting oligotrophic waters. The form without a posterior spine (tecta) is more common in eutrophic conditions. Obtained results agree with Ejsmont-Karabin (2012), showing that this indicator is not only one of the best indicators of trophic status but also an excellent indicator of the ecological status of stratified lakes.

Margalef’s index (*d*), which relates the number of species to the total number of individuals, was one of the two indicators of diversity tested in the study. In contrast to the Shannon Weaver index, it showed a highly statistically significant correlation with the parameters of pressure. To calculate Margalef’s index, the number of species and their abundance have to be taken into account, which means that the higher the index value, the better the ecological status of the lake. Obtained results show that an increase in the pollution level of the lake causes a decrease in the value of this index (see Fig. 2), which confirms the results of Haberman’s (1996, 1998) research, indicating that zooplankton species diversity decreases with increasing TP concentration.

Zooplankton abundance (NZOL; ind./L) is an indicator often used to assess the trophic status of lakes (Andronikova, 1996; Caroni & Irvine, 2010; Haberman & Haldna, 2014). It is well known that both the Rotifer and Crustacean abundance increase with an increase of trophy. The normalized values of NZOL index gradually decreased from 1 to 0.6 with increasing TP concentration, throughout the trophic gradient (see Fig. 2). This index is easy to calculate and is highly correlated with trophy (see Table 3).

The response of individual metrics to the increase in pressure expressed by TP concentration varied considerably (see Fig. 2). This was particularly evident in the case of the three metrics: CA/CY, IHTROT, and TECTA. CA/CY and IHTROT were most sensitive to a slight increase in TP concentration. The shape of the response curve of CA/CY metric is caused by displacement of Calanoida by Cyclopoida. Calanoida (mainly herbivores) occurs usually in oligotrophic environments where nanophytoplankton dominates (Hillbricht-Ilkowska, 1972). When conditions deteriorate, the domination is taken over by Cyclopoida, which prefers eutrophic conditions due to its ability to digest larger particles of food (Pace, 1986). IHTROT was also very sensitive to low TP concentrations (values decreased rapidly), while beyond 35 μgP/L of TP concentration, the decrease in the value became gradual. Due to the small body size of Rotifera, this group of zooplankton is released from the pressure of plankivorous fish, and thus, the population is regulated only by a bottom-up strategy (Ejsmont-Karabin, 2012). Changes in the community reflect a direct reaction to the enrichment of the waters with nutrients, and even a slight deterioration of the trophic conditions in reservoir causes a rapid increase in the share of species that prefer eutrophic conditions. Part of Rotifera preferring low-trophic waters are sensitive to the increase in eutrophication (similarly to Calanoida), and as the trophy increases, its abundance decreases. The reaction of this metric may result from the different sensitivities of individual indicator species to the trophic growth. Conversely, the TECTA metric was least sensitive to changes at low TP concentrations; however, its values decreased rapidly beyond 45 μg/L of TP concentration. The *tecta* form of *Keratella cochlearis* is absent in lakes of a very low trophy, while it is abundant in eutrophicated lakes (Ejsmont-Karabin, 2012). The morphological variability of *Keratella cochlearis* is probably determined by the abundance of phytoplankton and sestonu (Hillbricht-Ilkowska, 1972). This index is useful as a multimetric component for determining the border between good and moderate status, since *tecta* form does not occur in clean lakes (oligo- and mesotrophic). When the conditions in lakes deteriorate, the *tecta* form begins to appear in the zooplankton community and its abundance increases with an increase of trophy. The ZIPLAs multimetric index, with all of the above-mentioned components, enables the assessment of changes in the zooplankton community in the full trophic gradient.

ZIPLA_S_ index values decrease with increasing lake eutrophication. This index is most sensitive to the deterioration of lake conditions, i.e., increase in TP and decline of SD; however, it is less sensitive in lakes where phosphorus values exceed 70 µg TP/L (see Fig. 3). The ZIPLA_S_ differentiated between good and moderate status, which is crucial when developing biological methods in accordance with the WFD, indicating that this index is very sensitive to even a slight deterioration in lake water quality.

The zooplankton metrics presented above, which are components of the newly developed multimetric, are easy to calculate and do not require detailed knowledge of zooplankton species or the calculation of biomass according to complex formulas.

Additionally, one summer field campaign is sufficient to calculate a ZIPLA_S_ multimetric. The summer stagnation is the most stable period, when changes in the abiotic and biotic environmental conditions are slight. During this period, zooplankton communities are most diversified and attain the highest abundance level (Karabin, 1985). Single sampling during the summer season is cost-efficient and has a potential to be useful for routine monitoring of lakes located in Poland and temperate zones. Moreover, the identification of zooplankton species is much easier than that of phytoplankton. Additionally, zooplankton samples are easy to collect and can be taken during the phytoplankton field campaign. All of these features make zooplankton a cost-efficient indicator that cannot be replaced by sampling fish or phytoplankton. Another potential consideration is the use of a newly developed zooplankton index to replace the costly monitoring of ichthyofauna, which not only interferes with the structure of the fish population but may also be inaccurate in the case of Polish lakes, where the assessment based on ichthyofauna remains debatable due to the continuous stocking processes. Mills et al. (1987) claim that zooplankton size can provide information regarding both the ratio of predator to prey and the structure of the fish community.

The proposed ZIPLAs multimetric index can be considered as a useful tool for assessing the ecological status of Polish lakes. It can also be used to assess lakes with similar abiotic types in temperature zone, while the use of the index in other regions requires adaptation of the list of indicator species.

## Conclusions

Zooplankton is widely considered a central component of a pelagic food web in lakes. It is sandwiched between planktivorous fish (“top-down” control) and phytoplankton (“bottom-up” control), thus reflecting slight changes occurring in higher- and lower-trophic levels. As emphasized by Jeppesen et al. (2011), “Zooplankton has a strong indicator value, which cannot be covered by sampling fish and phytoplankton without a very comprehensive and costly effort.” (p. 279, abstact). The ZIPLA_S_ serves as a new tool for measuring the ecological status of lakes and can provide a useful way to monitor even minor changes in lake water quality, derived from anthropogenic pressure. The sampling method used in the development of ZIPLA_S_ is straightforward and cost-efficient compared to other biological methods and can be applied to other European stratified lakes in temperate zone. Results show that ZIPLA_S_ would be a valuable addition to the WFD system, among the rest of the biological elements.

## Data Availability

Data are available from the authors upon reasonable request.
